# Soluble immune checkpoints in cancer: production, function and biological significance

**DOI:** 10.1186/s40425-018-0449-0

**Published:** 2018-11-27

**Authors:** Daqian Gu, Xiang Ao, Yu Yang, Zhuo Chen, Xiang Xu

**Affiliations:** 1 Department of Stem Cell & Regenerative Medicine, State Key Laboratory of Trauma, Burn and Combined Injury, Daping Hospital and Research Institute of Surgery, Third Military Medical University, Chongqing, 400042 People’s Republic of China; 2 First Department, State Key Laboratory of Trauma, Burn and Combined Injury, Daping Hospital and Research Institute of Surgery, Third Military Medical University, Chongqing, People’s Republic of China

**Keywords:** Immune checkpoints, T cells, Soluble receptors and ligands, Immunotherapy, Cancer

## Abstract

Immune checkpoints play important roles in immune regulation, and blocking immune checkpoints on the cell membrane is a promising strategy in the treatment of cancer. Based on this, monoclonal antibodies are having much rapid development, such as those against CTLA-4 (cytotoxic T lymphocyte antigen 4) and PD-1 (programmed cell death protein 1).But the cost of preparation of monoclonal antibodies is too high and the therapeutic effect is still under restrictions. Recently, a series of soluble immune checkpoints have been found such as sCTLA-4 (soluble CTLA-4) and sPD-1 (soluble PD-1). They are functional parts of membrane immune checkpoints produced in different ways and can be secreted by immune cells. Moreover, these soluble checkpoints can diffuse in the serum. Much evidence has demonstrated that these soluble checkpoints are involved in positive or negative immune regulation and that changes in their plasma levels affect the development, prognosis and treatment of cancer. Since they are endogenous molecules, they will not induce immunological rejection in human beings, which might make up for the deficiencies of monoclonal antibodies and enhance the utility value of these molecules. Therefore, there is an increasing need for investigating novel soluble checkpoints and their functions, and it is promising to develop relevant therapies in the future. In this review, we describe the production mechanisms and functions of various soluble immune checkpoint receptors and ligands and discuss their biological significance in regard to biomarkers, potential candidate drugs, therapeutic targets, and other topics.

## Introduction

Immune checkpoints are molecules that can increase or decrease the signals of the immune system, and they are considered to be critical factors in treating infections, cancers and autoimmune diseases. Currently, immune checkpoint therapy is seen as a pillar of cancer therapy [1]. Among the different checkpoint therapies, those involving PD-1 and CTLA-4 may be the most effective. CTLA-4 is considered to be the first functional immune checkpoint, as it stops T cells in lymph nodes at the initial stage of naive T-cell activation, while the PD-1 pathway suppresses activated T cells at the later stages of an immune response, typically in peripheral tissues [2]. In clinical trials, the anti-CTLA-4 antibody and the anti-PD-1 antibody have shown tremendous promise against a wide spectrum of solid and hematological malignancies, significantly improving OS (overall survival) in newly diagnosed and heavily pretreated patients alike [3]. However, the influences of soluble receptors and ligands on immune regulation and cancer treatment have been less well studied. Soluble receptors and ligands, which are part of a family including full-length receptors and ligands, are produced by mRNA expression or by the cleavage of membrane-bound proteins and are found free in the plasma. These entities may play important roles in immune regulation via interactions between soluble receptors and full-length ligands or between soluble ligands and full-length receptors. For example, alternative splice variants of the human PD-1 and CTLA-4 genes have been identified, and sPD-1 can interfere with PD-L1/2 (programmed cell death ligand-1/2, also known as B7-H1/2):full-length PD-1 interactions, thereby blocking the negative signal imparted by the transmembrane form of PD-1 [4, 5]. Several studies have documented many types of soluble receptors and ligands that can be detected in the plasma in cancer, and the plasma levels are related to the severity of cancer.

Since previous studies suggested that soluble receptors and ligands should be considered therapeutic targets in cancer, we introduce some common therapeutic targets. We also review the production of these soluble receptors and ligands and discuss related clinical findings. We not only consider the significance of these receptors and ligands with regards to the prognosis and treatment in cancer but also consider their mechanisms of action. Finally, we conclude the use of immunotherapy based on these soluble molecules.

## State of immune checkpoints

Immune checkpoints can stimulate or inhibit signals in immune cells and regulate their functions; thus, the checkpoints play important roles in the maintenance of immune homeostasis [6]. For example, T cells need two signals for activation: binding of the TCR (T-cell receptor) and the MHC (major histocompatibility complex) and an interaction between costimulatory molecules [7]. On the contrast, PD-L1 expressed by some tumors works as a coinhibitory ligand with PD-1 to prevent T-cell activity [8].

### Categories of immune checkpoints

**Fig. 1:**
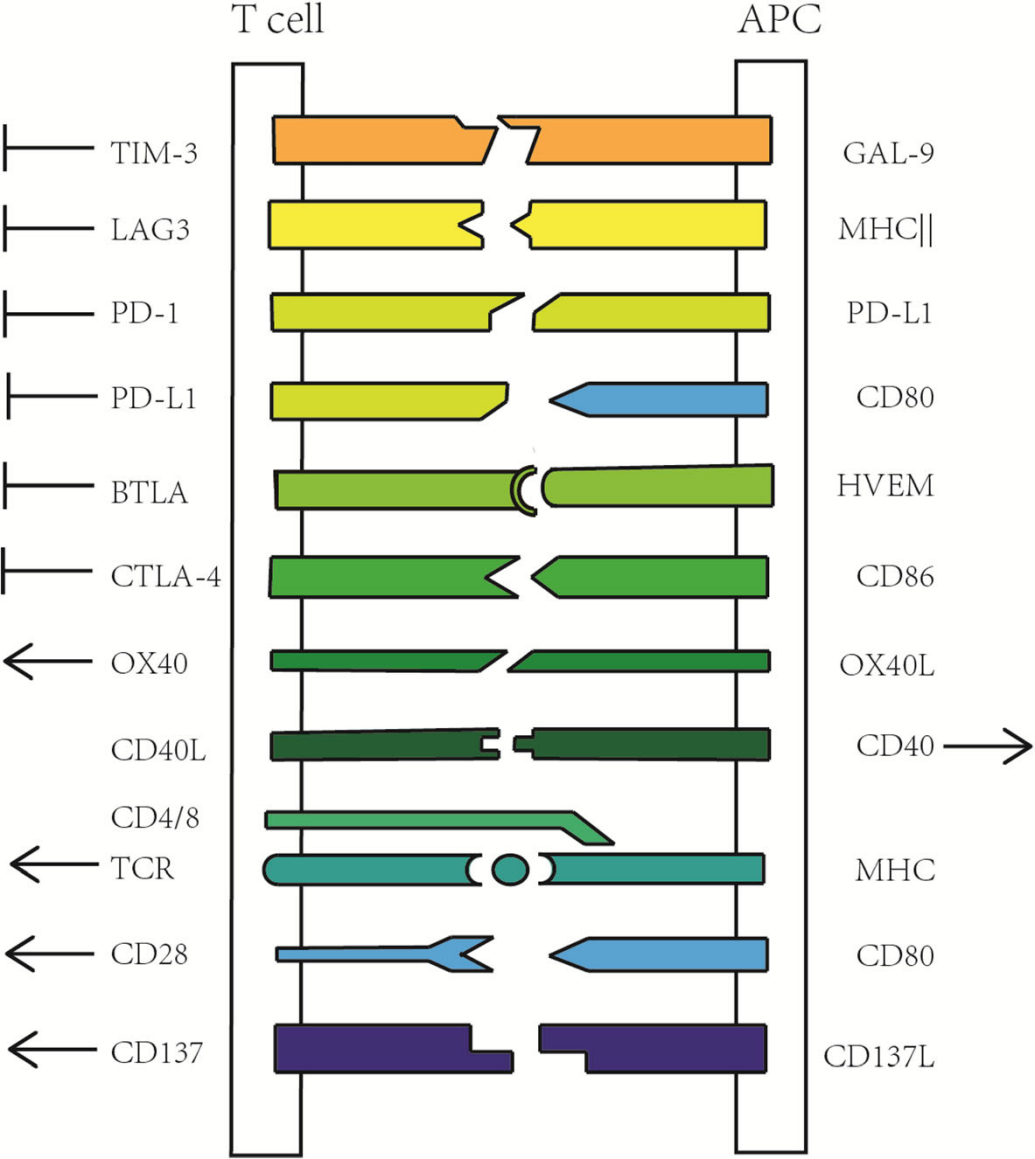
Various ligand-receptor interactions between T cells and APCs (generalized to include all karyocytes). TIM-3, LAG3, PD-1, PD-L1, BTLA, and CTLA-4 are coinhibitory molecules present on the surface of T cells. OX40, TCR, CD28, and CD137 can transfer stimulatory signals into T cells, and CD40 can receive stimulatory signals from CD40L on T cells

In the immune system, checkpoints can be divided into two groups: stimulatory molecules such as TCR/MHC and inhibitory molecules such as CTLA-4/CD80 or CD86 and PD-1/PD-L1. Increasing numbers of novel receptors and ligands have recently been found in the immune system. Some take part in costimulatory interactions, such as CD137L/CD137 and OX40L/CD40 [9], while others, such as HVEM/BTLA and MHCII/LAG3 [10], are involved in inhibitory interactions. Apart from these, other receptors have also become renowned for their unique functions. For example, GAL-9/TIM-3 can induce the inhibition of Th1 cell responses [11]. In Fig. 1, we summarize the various ligand-receptor interactions of immune checkpoints between T cells and APCs (antigen-presenting cells).

### Basic functions

Immune checkpoints can transfer signals between different immune cells, changing their activities and regulating the secretion of cytokines in response to the microenvironment. For example, when the TCR on Th1 cells combines with MHCII on APCs, the Th1 cells are activated and can secrete IL-2 and IFN-γ (interferon-γ) to enhance the antitumor effects [12] .

PD-1 and CTLA-4 are excellent examples of immune checkpoints. Unlike other members of the CD28 family, PD-1 can only transduce signals when crosslinked with BCR or TCR. Various studies have confirmed that PD-L1 and PD-L2 are expressed in cancer cells, T cells, macrophages (mostly M2 macrophages), myeloid DCs (dendritic cells), myeloid suppressor cells, stromal fibroblasts, and endothelial cells, suggesting that PD-1/PD-L1/PD-L2 can influence many cell types. The PD-1/PD-L1 pathway in the TME (tumor microenvironment) can inhibit the activation of effector T cells and promote the generation of Treg cells; this shows that PD-1-induced immune suppression may cause cancer cells to escape immune surveillance [13]. The blockade of this pathway in many therapeutic strategies can promote antitumor effects. CTLA-4 is expressed in T cells, B cells, NK cells, NKT cells, and DCs. CTLA-4 is homologous to CD28, but it has an approximately 100-fold higher affinity for both CD80 and CD86 than CD28. Similar to PD-1, CTLA-4 inhibits T cell activation by binding to its ligand [14]. Moreover, CTLA-4 also inhibits IL-2 production and influences naive CD4^**+**^ T cell differentiation. Both antibody blockade of CTLA-4 and genetic deletion of CTLA-4 induce the generation of Th17 cells and improve Th2 cell differentiation. In addition, CTLA-4 can control not only T cell effector functions but also B cell responses by regulating the functions of T follicular helper cells and T follicular regulatory cells [15]. Though the critical role of CTLA-4 in controlling T cell activation and tolerance is well known, how CTLA-4 exerts its inhibitory effects remains unclear.

### Increased significance

With the development of immunotherapy for cancer and other diseases, the demand for identifying immune checkpoints is growing. The FDA has approved a series of antibodies targeting these checkpoints. In addition, some novel strategies based on the principle of immune checkpoints have been developed. For example, a combination of synergistic immune checkpoint blockade and targeted therapy is used to treat metastatic melanoma [16]. Because of the need for the endogenous molecule used for therapy, the formation mechanisms and functions of soluble receptors and ligands produced by human body, such as sPD-1, are being explored [17].Due to the rapid development of Immune checkpoint therapy, it will likely become the most effective way to fight cancer, although this therapy still has some limitations, such as a lack of power in the TME [18].

## Soluble immune checkpoints

In addition to the receptors and ligands of immune checkpoints on cell membrane, a series of soluble immune checkpoints have also been analyzed, and their plasma levels have been measured. These checkpoints play an important role in immune regulation, are involved in the development and prognosis of cancer, and are considered to be potential biomarkers and therapeutic targets. A summary of the information gathered on the soluble immune checkpoints is shown in Table 1.

**Table 1:** Basic information about the soluble receptors and ligands

Soluble receptors/ligands	Structure	Cell source	Production	Ligands/receptors	Main functions	references
sPD-1	Monomer	PBMCs (peripheral blood mononuclear cells)	mRNA expression	PD-L1/2	1. Block PD-L/PD-1 interactions 2. Activate CD8^**+**^ T cells	[4, 19, 20, 23, 24]
sPD-L1	Unknown	Mature DCs	Possible cleavage of membrane-bound proteins	PD-1	1. Combine with PD-1 2. Inhibit T-cell responses	[31, 32, 44]
sPD-L2	Unknown	Activated leukocytes	mRNA expression	PD-1	Unknown	[45]
sCTLA-4	Monomer	Monocytes/immature DCs/Treg cells	mRNA expression	CD80/CD86	Inhibit T-cell responses	[46, 47, 51, 52]
sCD80 (sB7–1)	Homodimer	Unstimulated monocytes/B cells	mRNA expression	CTLA-4 /CD28	Inhibit PD-1/PD-L1 pathway	[53, 54, 56, 59, 60]
sCD86 (sB7–2)	Monomer	Resting monocytes/DCs	Cleavage of membrane-bound proteins/mRNA expression	CTLA-4	Inhibit T-cell responses	[65, 69]
sB7-H3	Unknown	Monocytes/ DCs/activated T cells	Cleavage of membrane-bound proteins/mRNA expression	B7-H3R	Promote IL-8 and VEGF expression	[70, 71, 77]
sCD137 (s4-1BB)	Unknown	Activated PBMCs	Cleavage of membrane-bound proteins/mRNA expression	CD137L	Inhibit CD137/CD137L pathway	[78, 79, 82]

For example, sPD-1 has been reported to be a monomer, and it can be produced by PBMCs through mRNA expression. Moreover, sPD-1 can combine with PD-L1 and PD-L2, thus blocking PD-L/PD-1 interactions and somehow activating CD8**+** T cells. Some soluble receptors can be produced by the cleavage of membrane-bound proteins or by both the cleavage of membrane-bound proteins and mRNA expression

## sPD-1

### Production

The sPD-1 was reported to be a monomeric protein [19]. Christian Nielsen et al. found that sPD-1 is generated from mRNA expression. Four alternatively spliced PD-1 mRNA transcripts—PD-1△ex2, PD-1△ex3, PD-1△ex2,3, and PD-1△ex2,3,4—were described apart from the full-length PD-1. These variants are generated by splicing out exon 2; exon 3; exons 2 and 3; and exons 2, 3, and 4, respectively. In contrast to the other transcripts, which do not have obvious biological functions, PD-1△ex3 is the soluble isoform of PD-1 and increases following the activation of PBMCs [4].

### Prognosis

One study on sPD-1 found that its existence in tumor tissue promotes tumor-specific immunity, and in immunocompetent mice, a striking degree of immune cell infiltration was observed on local tumor, that was thought to be related to prolonged survival [20]. In addition, in a study on NSCLC (non-small cell lung cancer), elevated sPD-1 was found in 34% of patients receiving erlotinib and these patients experienced prolonged progression-free and OS [21]. Notably, in a cohort of 2903 HBV patients, higher sPD-1 level appears to be associated with an increased risk of HCC (hepatocellular carcinoma) [22].

### Biological mechanism

sPD-1 can inhibit all three PD-L/PD-1 interactions: PD-L1/CD80, PD-L1/PD-1, and PDL2/PD-1 [23]. Osama et al. found that expressed sPD-1 blocks PD-L1/PD-1 interactions, which explains the inhibition of tumor growth after local gene transfer of sPD-1 in tumor inoculation sites [20]. Researchers have also used adenovirus to transduce the thymidine kinase gene and sPD-1 into tumors, which causes tumor regression by upregulating the activation of CD8^**+**^ T cells [24]. Moreover, in research on cancer treatments using a combination of the HSP70 vaccine and sPD-1, it has been found that sPD-1 can not only block PD-L1 but also reduce the expression of IL-10 gene, a negative regulatory gene [25]. Fibronectin CH50 has been demonstrated to increase the activity of macrophages, and in vivo studies have demonstrated that a sPD-1-CH50 recombinant peptide increases the cytolytic activity of both macrophages and cytotoxic T lymphocytes, especially towards PD-L1-positive tumor cells. This effect is due to the increased production of inducible nitric oxide synthase, TNF-α (tumor necrosis factor-α), and IFN-γ [26]. In addition, the combination of 4-1BBL and sPD-1 decreases the expression of IL-10 and TGF-β in treated mice, thus inducing the expression of IL-2 and IFN-γ and the accumulation of CD8+ T cells in the TME. Furthermore, one research team constructed a recombinant eukaryotic expression plasmid encoding sPD-1 to investigate the effects of a blockade of sPD-1/PD-L1 interaction, the antitumor response of T cells to sPD-1 and the local therapeutic effect of sPD-1 on mouse hepatocarcinoma. After coculturing sPD-1 with tumor cells (H22 cell line) and spleen lymphocytes, the group demonstrated a dual effect of sPD-1: an enhancement of the immune response through interaction with immune cells such as DCs and a blockade of PD-L1 on tumor cells [27].

**Fig. 2:**
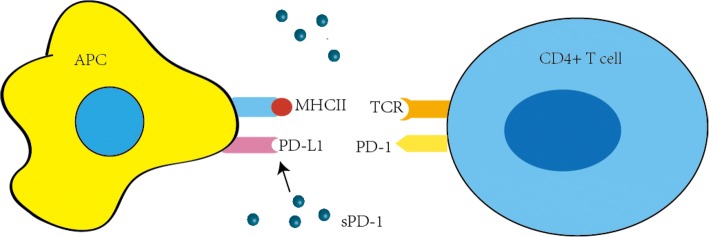
The role of sPD-1 between APCs and T cells. Some studies have demonstrated that when sPD-1 binds to PD-L1 in APCs, it can induce a reverse signal that acts on APCs and inhibits T cell function. However, other experiments have found that the existence of PD-L1/sPD-1 enhances the function of T cells. T cells can be activated with the anti-PD-L1-mAb

Above all, we can speculate that sPD-1 can interact with PD-L1 and prevent PD-1 from combining with PD-L1; in other words, sPD-1 competes with PD-1 in vivo [28]. However, Harmjan Kuipers, et al. reported a different phenomenon. They cocultured DCs and T cells with sPD-1 and observed an inhibition of T cell proliferation and IL-2 production. They speculated that reverse signaling may take place when sPD-1 binds to PD-L1 on DCs **(see** Fig. 2**)** [29].

### Treatment methods

Thus far, some treatments using sPD-1 have been tested in mice, but the use of this treatment has not been reported in humans. However, mouse models can provide insight to improve future antitumor treatments for humans. It has been reported that when the HSP70 vaccine and sPD-1—which are used to treat cancer and to block PD-L1, respectively—are combined, mice experience a significantly prolonged survival time compared to mice treated with HSP70 or sPD-1 separately [25]. In China, researchers used naked plasmids to deliver sPD-1-CH50 into tumor inoculation sites and found that sPD-1-CH50 stimulates more effective antitumor activity than sPD-1, CH50 or sPD-1/CH50, which shows that the recombinant protein sPD-1-CH50 can be used as a therapeutic strategy after the surgical removal of tumors [26]. Moreover, after researchers administered sPD-1 DNA with the human papilloma virus-16 E7 DNA vaccine to mice, E7-specific CD8^**+**^ T cell responses were significantly enhanced, resulting in potent antitumor effects against E7-expressing tumors and causing a significant increase in the survival rate up to 75%; this suggests a role for sPD-1 DNA as a genetic adjuvant and for prophylactic antitumor treatment [23]. In mice with H22 hepatocarcinoma, naked plasmid of 4-1BBL and sPD-1 were injected for local gene transfer; ultimately, this treatment completely eradicated tumors from mice with small numbers of preexisting tumor cells and eradicated tumors from 60% of individuals with larger numbers of preexisting tumor cells [30].

## sPD-L1

### Production

sPD-L1 can be produced and released by both tumor cells and activated mature DCs, while immature DCs, macrophages, monocytes and T cells are refractory to releasing sPD-L1 [31]. sPD-L1 is detectable in human serum, and its concentration increases with age; furthermore, it has been reported that increased MMPI (matrix metalloproteinase inhibitor) reduces the production of sPD-L1 in PD-L1 transfected cells, suggesting that sPD-L1 may be produced by the proteolytic cleavage of membrane-bound proteins [32]. PD-L1 is encoded by the CD274 gene which comprises seven exons on chromosome 19 in mice and on chromosome 9 in humans. However, evidence that sPD-L1 can be produced by alternative splicing has not been found [33].

### Prognosis

In a French multicenter randomized phase III clinical trial, researchers found that the levels of sPD-L1 in the plasma of patients with DLBCL (diffuse large B-cell lymphoma) were much higher than healthy humans. These patients were treated with high-dose chemotherapy and rituximab. Then, patients with elevated sPD-L1 experienced poorer prognosis, with a 3-year OS rate of 76% versus 89% in healthy individuals. When patients were in CR (complete remission), their sPD-L1 levels returned to normal [34]. In a clinical study about malignant melanoma, early changes in sPD-L1 levels after checkpoint blockade treatment did not correspond with benefit. However, rise in sPD-L1 after 5 months of treatment correlated with partial responses in ipilimumab-treated patients. And rise in sPD-L1 after pembrolizumab treatment was also associated with partial responses, and high pre-treatment levels were associated with disease progression [35]. For nivolumab-treated patients with NSCLC, lower basal plasma levels of sPD-L1 were associated with better clinical benefit, but the changes during treatment were still vague [36]. In another study on 141 patients with HBV-related HCC, circulating PD-L1 expression was closely related to intratumoral PD-L1 expression and PD-1/PD-L1 expression was associated with tumor size, blood vessel invasion and BCLC (Barcelona Clinic Liver Cancer) stage. Moreover, patients with higher expression of circulating PD-L1 and PD-1 had shorter OS and tumor-free survival times than those with lower expression. These results show that patients with higher sPD-1 and sPD-L1 levels have a worse prognosis [37]. In a study on NKTCL (natural killer/T-cell lymphoma), patients with a high concentration of serum sPD-L1 (≥3.4 ng/ml) or with a high percentage of PD-L1 expression in tumor specimens (≥38%) responded poorly to treatment and exhibited markedly worse survival than patients with lower concentrations or lower percentages of expression. Furthermore, a high concentration of serum sPD-L1 and a high percentage of PD-L1 expression in tumor specimens can be independent adverse prognostic factors in patients with stage I~II NKTCL [38]. Similar to the study above, studies by both Wang and Huang’s teams found that the overall response rate to treatment was higher in low sPD-L1 patients than in high sPD-L1 patients with MM (multiple myeloma), indicating a poorer prognosis in patients with higher levels of sPD-L1 (> 2.783 ng/mL) [39, 40]. In patients with oral squamous cell carcinoma, the increased expression of sPD-L1 has also been found to be associated with poor prognosis [41]. Similarly, in HL (Hodgkin Lymphoma), sPD-L1 levels are positively correlated with clinical stage [42]. However, the phenomenon in advanced gastric cancer seemed to be contradictory to the above researches, in which adenocarcinoma patients with higher PD-L1 expression had much better prognosis and less lymph node metastasis than low-expression patients [43].

### Biological mechanism

A study on the role of sPD-L1 found that IFN-γ secretion by CLL (chronic lymphocytic leukemia) T lymphocytes decreases significantly in the presence of sPD-L1. Conversely, treatment with an anti-PD-L1 antibody leads to a significant increase in IFN-γ secretion by CLL T lymphocytes [44], and coincubation of CD4+ or CD8+ T cells with sPD-L1-producing cells and mDC-derived sPD-L1 induces T cells to undergo apoptosis [31]. As sPD-L1 spreads throughout the body via the blood and lymphatic circulation, it exerts a widespread inhibitory effect by interacting with cell surface receptors such as membrane-bound PD-1 [44].

## sPD-L2

Two novel human PD-L2 splice variants have been identified. In the major variant, exon 3 is cut off, and the protein product lacks the IgC-like domain and is shorter in the extracellular region. Although the other variant is also generated by cutting off exon 3, the acceptor site for that variant is 5 bp downstream of the canonical acceptor site. This second variant also has a frameshift such that its protein product lacks the transmembrane domain and is secreted in a soluble form, which is thought to be sPD-L2. These findings suggest that sPD-L2 expression may be controlled by posttranscriptional regulation through alternative splicing [45].

## sCTLA-4

### Production

Although the prominent source of sCTLA-4 is Treg cells, the sCTLA-4 transcripts have also been detected in both monocytes and immature DCs [46]. Magistrelli et al. identified an additional splice variant named CTLA-4delTM that lacks both the transmembrane and intracellular domains. The splice variant, derived from the deletion of exon 2 (which encodes the transmembrane domain and the cytoplasmic tail of CTLA-4), is thought to be translated into sCTLA-4. Furthermore, CTLA-4delTM can be produced as a soluble monomer [47].

### Prognosis

There have been only a few reports on sCTLA-4 levels in the serum of patients with cancer. In a study, for ipilimumab–treated patients with melanoma, those who could respond to the treatment had higher serum levels of sCTLA-4 (mea*n* = 2417 pg/mL) and experienced longer OS [48]. In addition, recent analyses of primary melanoma cell lines have demonstrated that the cells can secrete detectable levels of sCTLA-4, supporting the relevance of this molecule in cancer. And in B-ALL (B-cell acute lymphoblastic leukemia) patients, the correlation between sCTLA-4 and neoplastic B cells was apparently negative [49]. Furthermore, it has been shown that sCTLA-4 is expressed by malignant B cells, at least in pediatric ALL patients, and the release of sCTLA-4 from acute lymphoblastic leukemia cells may constitute a strategy for immune-surveillance escape [50].

### Biological mechanism

Analysis of human T cells in vitro has shown that sCTLA-4 secretion can increase during immune responses and has potent inhibitory properties, as isoform-specific blockade of sCTLA-4 significantly increases Ag(antigen)-driven proliferation and cytokine (IFN-γ, IL-17) secretion [46]. Similar to full-length CTLA-4, sCTLA-4 can bind to B7 costimulatory ligands on APCs to prevent B7 from combining with the costimulatory receptor CD28 in T cells, thus inhibiting T-cell responses. Furthermore, sCTLA-4 can neutralize the anti-CTLA-4-mAb in vivo. Indeed, the inhibition of sCTLA-4 with anti-sCTLA-4-mAb induces significant increases in antigen-specific immune responses both in vitro and in vivo. In human peripheral blood mononuclear cell responses, the selective blockade of sCTLA-4 activates the proliferation of CD8^**+**^ and CD4^**+**^ T cells and promotes increased cytokine secretion, most notably the secretion of IFN-γ, which in turn enhances antitumor effects [51]. As is the case for sPD-1, the affinity of sCTLA-4/CD80 has not been reported. However, CTLA-4 binds to CD80 and CD86 with Kd values of 0.2 μM and 2.6 μM respectively, which are approximately 10-fold lower than the Kd values for the binding of CD28 to CD80 and CD86 (4 μM and 20 μM, respectively) [52].

### Treatment methods

Thus far, there have been few studies on the implications of sCTLA-4 in cancer treatment. With regards to anti-CTLA-4-mAbs, it is interesting that selective blockade of sCTLA-4 can not only enhance antigen-specific CD4^**+**^ and CD8^**+**^ T-cell responses but also exert functional antitumor activity without requiring an interaction with full length CTLA-4 in a murine model of melanoma [51].

## sCD80

### Production

CD80 is a costimulatory factor mainly expressed on the surface of activated monocytes, B cells and DCs. Kakoulidou et al. found that a spliced form, sCD80, is expressed in unstimulated monocytes and B cells. sCD80 lacks the transmembrane domain and can bind to recombinant CD152-Ig, CD28-Ig and activated T cells [53]. sCD80 is thought to be a homodimer based on an analysis of its structure [54].

### Prognosis

In one clinical study, the sCD80 levels in the majority of patients with AML (acute myeloid leukemia) (13/17) and MM (11/12) were normal. However, significantly elevated levels were detected in CLL and MCL (mantle cell lymphoma) patients. Furthermore, increased sCD80 levels in CLL patients were significantly associated with poor prognosis and were accompanied by low platelet and hemoglobin levels with elevated WBC counts and the expression of CD38 [55].

### Biological mechanism

Kakoulidou et al. reported that recombinant sCD80 has immunomodulatory effects, as shown by its inhibition of the mixed lymphocyte reaction and T-cell proliferation; they speculated that the preferential binding of sCD80 to CD152 is responsible for the inhibitory reaction [53]. In contrast, Wei et al. posited that soluble B7-IgG can bind to CTLA-4 on activated T cells with a high affinity, blocking the negative signals triggered by sCD80—which is different from the response triggered by membrane-bound CD80 [56]. Furthermore, Sturmhoefel et al. found that soluble B7-IgG can induce T-cell proliferation in therapy for established tumors [57]. More concrete mechanisms have since been studied. For example, one study found that a soluble form of CD80, CD80-Fc (in which the extracellular domains of human or mouse CD80 are fused to the Fc domain of IgG1), increased the production of IFN-γ by PD-1^**+**^ activated T cells more effectively than antibodies to PD-1 or PD-L1, possibly by neutralizing PD-L1 or costimulating with CD28 [58]. Suzanne et al. and Samuel et al. achieved similar results, finding that CD80-Fc could sustain IFN-γ production by both human and murine PD-1^**+**^ activated T cells in the presence of PD-L1^**+**^ human or mouse tumor cells, respectively. They also found that CD80-Fc simultaneously inhibited PD-L1/PD-1-mediated immune suppression [59, 60].

### Treatment methods

In a preclinical study, the CD80-Fc was used in combination with Treg cell depletion, which dramatically controlled the colon tumor size and enhanced antitumor activity. Furthermore, the mice in the study exhibited immunologic memory since they can reject subsequent implants in rechallenge experiments [61]. In another study, the CD80-Fc fusion protein gene was delivered to tumor cells in vivo in the context of an oncolytic replication-competent herpes simplex virus [62]. However, Zhou et al. described a nonviral intramuscular gene transfer method to deliver this therapeutic protein, after which muscle tissue can exert immune costimulatory effects for cancer therapy by producing the protein in large quantities. This gene transfer method has also been used as an adjuvant therapy for DNA vaccination [63]. Combination therapy has also been considered. For example, Yasushi et al. combined IL-12, IL-18, and sCD80 with oncolytic herpes simplex virus-1 vectors in a treatment and showed strong antitumor activity [64].

## sCD86

### Production

sCD86 is produced by resting monocytes in human beings. Jeannin et al. demonstrated that the sCD86 detected in human serum can be generated by the translation of the CD86△TM mRNA, which is characterized by the deletion of the transmembrane domain. And sCD86 is formed as a monomer [65].

### Prognosis

Hock et al. reported that the plasma of a proportion of examined leukemia patients contained elevated levels of sCD86, but the sCD86 levels were not directly related to CRP (C-reactive protein)levels, suggesting that increases in sCD86 are not solely related to a broad inflammatory response. Furthermore, no relationship between sCD86 levels and prognosis was found [66]. In another study, levels of sCD86 were elevated (> 2.32 ng/mL) relative to normal donors in 25% of patients with AML and in 27% of patients with MDS (myelodysplastic syndrome). In addition, compared to AML patients with normal sCD86 levels, patients with AML who had elevated sCD86 levels experienced significantly lower CR rates and poorer survival. However, the correlation between sCD86 levels and CR rates or survival rates in patients with MDS was not found [67].

In 299 patients from the UK Medical Research Council myeloma VIth trial, Hock et al. reported that serum levels of sCD86 were significantly elevated. They also found that elevated sCD86 levels were associated with significantly shorter survival (median = 22 vs. 51 months) and event-free survival times (median = 14 vs. 31 months) in ABCM + P patients (patients receiving adriamycin, carmustine, cyclophosphamide, and melphalan with prednisolone), which suggested that sCD86 may be an important prognosis marker in at least some myeloma treatment groups [68].

### Biological mechanism

There have been few reports on the function of sCD86 in serum. Juan et al. found that co-delivery of sCD86 downregulated the immune response to a DNA vaccine, suggesting that sCD86 may bind to CTLA-4 to transfer a negative signal to T lymphocytes [69].

## sB7-H3

### Production

Zhang et al. demonstrated that sB7-H3 is released by monocytes, DCs, activated T cells, and various mB7-H3^**+**^ (membrane B7-H3^**+**^) cells but not by mB7-H3^**−**^carcinoma cells. After the addition of MMPI, the release of sB7-H3 from cells is blocked, indicating that the release of sB7-H3 from B7-H3 on the cell surface is mediated by a matrix metalloproteinase [70]. Moreover, Chen et al. found that sB7-H3 is also generated by alternative splicing of mRNA [71].

### Prognosis

In one study conducted by a single center, the expression of sB7-H3 and sPD-L1 in the CSF (cerebrospinal fluid) of the patients with glioma was higher than the patients with a moderate traumatic brain injury. Furthermore, the expression of B7-H3 and PD-L1 in CSF and tumor tissues was related to the glioma grade [72]. In clear cell renal cell carcinoma, both the serum level of sB7-H3 and sIL-2R (soluble IL-2R) are significantly correlated with the clinical stage, and the level of sB7-H3 shows a positive correlation with sIL-2R [73]. In a study, sB7-H3 concentrations were significantly higher in patients with ESHCC (early-stage hepatocellular carcinoma) than in cirrhotic patients (60.79 ± 19.45 ng/mL vs. 32.33 ± 11.52 ng/mL). Furthermore, high levels of sB7-H3 were correlated with poor clinical outcomes [74]. Chen et al. measured the expression of sB7-H3 in NSCLC-derived MPEs (malignant pleural effusions) and found that the median value of sB7-H3 in 52 MPEs was higher than that in 47 NPEs (nonneoplastic pleural effusions). Moreover, the levels of MPE-derived sB7-H3 were correlated with smoking status, primary tumor size (T factor), regional lymph node dissemination (N factor) and distant metastasis (M factor) in NSCLC patients, suggesting that increased sB7-H3 in MPEs is correlated with the TNM stage of NSCLC [75].

### Biological mechanism

sB7-H3 can bind to the B7-H3R (B7-H3 receptor) on activated T cells, showing that sB7-H3 is functional [70]. In Chen et al.’s study, T cell proliferation was significantly inhibited in the presence of sB7-H3 compared to the control group, and sB7-H3 significantly reduced the levels of both IL-2 and INF-γ in the culture supernatants compared to the levels in the control group, suggesting that sB7-H3 can negatively regulate T cell responses [71]. Sun et al. found that sB7-H3 can induce macrophages to increase the expression of MMR (macrophage mannose receptor) and IL-10 and decrease the expression of HLA(human leukocyte antigen)-DR and IL-1β in vitro, which can switch the macrophage phenotype from M1 to M2 [76]. Xie et al. observed that sB7-H3 was highly expressed in mB7-H3^**+**^ pancreatic carcinoma cells. Additionally, sB7-H3 promoted IL-8 and VEGF expression by first increasing TLR4 expression and then activating NF-κB signaling, which facilitated the formation of nascent blood vessels to help the cancer cells invade and metastasize [77].

## sCD137

### Production

Similar to murine sCD137, human sCD137 is generated by alternative mRNA splicing [78]. One study found that sCD137 can be generated by PBMCs; notably, the expression of sCD137 in lymphocytes requires strong activation, and the levels of sCD137 negatively correlate with lymphocyte proliferation and positively correlate with the degree of activation-induced cell death caused by mitogen overstimulation [79].

### Prognosis

According to a small single-center study, patients with colon cancer have significantly higher plasma levels of sCD137 than patients with rectal cancer (3931 ± 1268 pg/ml vs. 1194 ± 581 pg/ml). Interestingly, the levels of sCD137 and sCD137L are significantly correlated, indicating that divergent mechanisms might be involved in the pathogenesis of colorectal cancer [80]. Enhanced levels of sCD137 can be detected in the sera of patients with leukemia and lymphoma, and high sCD137 levels are strongly associated with CLL. However, why sCD137 is present in only a proportion of patients and whether sCD137 levels correlate with other parameters—such as disease stage, disease progression or therapeutic success—remain unclear [81].

### Biological mechanism

Labiano et al. induced tumor cells to generate sCD137 with hypoxia and demonstrated that tumor-secreted sCD137 prevents the costimulation of T lymphocytes by preventing the interaction of CD137L with the transmembrane forms of CD137 expressed on T lymphocytes [82].

### Treatment methods

In one study, breast cancer cells were treated with sCD137 in combination with SAHA (suberoylanilide hydroxamic acid), and the synergistic cytotoxic effect was enhanced, suggesting that a combination of SAHA and sCD137 could be a potential cancer therapy [83].

## Conclusions and future perspectives

The natural soluble forms of receptors and ligands are important components of immune regulation, although their definitive mechanisms of action have not been determined. In this review, we selected sPD-1, sPD-L1, sPD-L2, sCTLA-4, sCD80, sCD86, sB7-H3 and sCD137 for analysis. All of these molecules may play important roles in cancer. Many studies regarding these entities are ongoing, and the relevance of soluble receptors and ligands to various diseases is becoming increasingly apparent. As soluble molecules, their serum and tissue levels can be easily detected. These molecules may also be critical factors for evaluating the severity and prognosis of cancer and many other diseases since most patients experience changes in their levels (See Table 2); in addition, some soluble molecules have been reported to be predictive markers for the benefit of target therapy (See Table 3). In immunotherapy, immunogenicity of checkpoint inhibitors is still a severe problem, and the detection of anti-drug antibodies is still equated as a main way to measure immunogenicity [84]. According to the characteristics of soluble receptors, it is probable for them to neutralize the effect of monoclonal antibodies. Furthermore, whether they are included or paly important role in the hypersensitivity reactions during therapy is also unknown, since the interaction and changes of levels of these molecules are complicated. Thus, it is hopeful but there is a long way to find applicable soluble molecules to predict immunogenicity. Moreover, their exact functions are still unclear. Thus far, studies have developed methods to assess some of these proteins, such as sPD-1 and sCTLA-4. Thus, we can use these technologies for further research. In addition to detecting the proteins, some researchers have successfully mediated their serum levels to regulate the human immune system, suggesting that such manipulations can potentially be used in cancer treatment. Based on limited experimental and clinical findings, these soluble receptors and ligands can be novel therapeutic targets. Although it has been established that the concentrations of soluble receptors can influence the activation of APCs and T cells, the specific relevance of these factors is still unknown; nevertheless, we can use antibodies such as anti-PD-1-mAb and anti-CTLA-4-mAb to block these targets and neutralize their various functions in the progression of diseases. However, it may be necessary-yet difficult-to find more specific antibodies to precisely mediate these targets since current antibodies cannot distinguish between full-length receptors and soluble receptors. Although it will be some time before the precise regulatory roles of these soluble receptors and ligands are illuminated, it is imperative that they are considered in the formation of strategies for immunotherapy.

**Table 2:** Serum/plasma levels of soluble receptors and ligands detected in different types of diseases and their correlations with prognosis or outcomes

Soluble receptor/ligand	Related diseases	Number of patients	Type of analyses	Serum/plasma levels	Prognosis/outcomes	References
sPD-1	HCC	*n* = 126	Multivariate	+	High levels of viral load and sPD-1 associated with increase in risk of HCC	[22]
	RA (Rheumatoid arthritis)	*n* = 95	\	+	\	[85]
		*n* = 82	\	–	\	[86]
	ITP (Immune thrombocytopenia)	*n* = 67	\	–	\	[87]
	HCV (Hepatitis C virus) infection	*n* = 63	\	+	\	[88]
	AA (Aplastic anemia)	*n* = 80	\	+	\	[89]
	Sepsis	*n* = 112	Multivariate	+	High levels associated with higher 28-day mortality	[90]
	ARDS (Acute respiratory distress syndrome)	*n* = 10–13	\	+	\	[91]
sPD-L1	DLBCL	*n* = 288	Multivariate/ univariate	+	Elevated sPD-L1 experienced a poorer 3-year OS	[34]
	NKTCL	*n* = 77	Multivariate/ univariate	+	High concentration associated with shorter survival	[38]
	Oral squamous cell carcinoma	*n* = 82	Univariate	+	Higher levels associated with lower tumor cell differentiation	[41]
	HL	*n* = 108	Multivariate	+	Higher levels associated with shorter progression-free survival	[42]
	CE (Cystic echinococcosis)	*n* = 51	\	+	\	[44]
	AR (Allergic rhinitis)	*n* = 90	Univariate	–	Lower levels associated with lower disease severity	[92]
	T1DM (Type 1 diabetes mellitus)	*n* = 176	\	–	\	[93]
	T2DM (Type 2 diabetes mellitus)	*n* = 125	Univariate	+	Higher levels associated with increased severity of diabetic atherosclerotic macrovascular diseases	[94]
sPD-L2	SSc (Systemic sclerosis)	*n* = 91	Univariate	+	Higher levels associated worse pulmonary fibrosis	[95]
sCTLA-4	B-ALL	n = 80	\	+	\	[50]
	RA	*n* = 56	Univariate	+	Higher levels associated with higher disease activity	[96]
	Chronic hepatitis	*n* = 81	\	+	\	[97]
	T1DM	\	\	+	\	[98]
	SpA (Spondyloarthropathies)	*n* = 165	Univariate	+	Higher levels associated with higher disease activity	[99]
sCD80	CLL	*n* = 34	Univariate	+	Higher levels associated with poorer prognosis	[55]
	RA	n = 56	Univariate	+	The levels did not correlate with disease activity	[96]
	Acute asthma	*n* = 16	\	+	\	[100]
sCD86	Leukemia	n = 24	\	+	\	[66]
	AML	*n* = 57	Multivariate	+	Higher levels associated with shorter survival	[67]
	Myeloma	*n* = 299	Multivariate/ univariate	+	Higher levels associated with shorter survival in ABCM + P patients	[68]
	RA	*n* = 35	\	+	\	[101]
	SLE (Systemic lupus erythematosus)	*n* = 79	\	+	\	[102]
	Acute asthma	n = 16	\	+	\	[100]
		*n* = 68	\	+	Higher levels associated with acute asthma exacerbation	[103]
sB7-H3	Glioma	*n* = 78	Univariate	\	Higher levels associated with high-grade glioma	[72]
	ESHCC	*n* = 149	Multivariate/ univariate	+	Higher levels associated with lower survival rate	[74]
	Sepsis	*n* = 27	Univariate	+	Higher levels associated with a poor outcome	[104]
	Chronic prostatitis	n = 91	\	\	\	[105]
	MS (Multiple sclerosis)	*n* = 32	Univariate	–	Lower levels associated with a poorer outcome	[106]
	CHB (Chronic HBV infection)	*n* = 136	Univariate	+	Increased levels associated with the progression of liver cirrhosis	[107]
	SLE	n = 78	Univariate	–	Higher levels associated with higher disease activity	[108]
sCD137	Colon cancer	*n* = 76	\	+	\	[80]
	Leukemia and lymphoma	*n* = 173	\	+	\	[81]
	Acute pancreatitis	*n* = 41	Univariate	+	Higher levels associated with a poor outcome	[109]
	ACS (acute coronary syndrome)	*n* = 180	Multivariate	+	Higher levels associated with increased risk for adverse cardiovascular events	[110]
	Multiple sclerosis	*n* = 26	Univariate	+	Higher levels associated with higher disease activity	[111]
	RA	*n* = 30	Univariate	+	Higher levels associated with higher disease severity	[112]
	Acute atherothrombotic stroke	n = 27	\	+	\	[113]

Such studies supply information for the formulation of therapeutic strategies. The type of analyses refers to the way of analysis of the associations between levels and prognosis or outcomes. + means higher than controls; − means lower than controls; \ in serum/plasma levels means no significant difference between patients and controls, \ in types of analyses and prognosis/outcomes means no data found

**Table 3:** Biomarkers for clinical outcomes under target therapy for cancer patients

Biomarker	Cancer	Number of patients	Treatment	Level changes during treatment	Outcomes	References
sPD-1	NSCLC	*n* = 38	Erlotinib	Uncertain	Higher treatment levels associated with prolonged progression-free and overall survival	[21]
sPD-L1	DLBCL	*n* = 288	High-dose chemotherapy + rituximab	Uncertain	High pre-treatment levels associated with poorer prognosis	[34]
	Malignant melanoma	*n* = 251	Ipilimumab (±bevacizumab or sargramostim), pembrolizumab	Uncertain	High pre-treatment levels associated with disease progression	[35]
	NSCLC	*n* = 39	Nivolumab	Uncertain	Lower basal plasma levels associated with better clinical benefit	[36]
sCTLA-4	Melanoma	*n* = 14	Ipilimumab	Not mentioned	Higher sCTLA4 in responders, associated with longer OS	[48]

Such studies supply information for the formulation of diagnostic tools. Uncertain means the levels may be as same as control or be higher or lower than control in the study; not mentioned means no data found in the study

## Abbreviations

AA: Aplastic anemia
AML: Acute myeloid leukemia
APCs: Antigen-presenting cells
AR: Allergic rhinitis
ARDS: Acute respiratory distress syndrome
B7-H3R: B7-H3 receptor
B-ALL: B-cell acute lymphoblastic leukemia
BCLC: Barcelona clinic liver cancer
CE: Cystic echinococcosis
CHB: Chronic HBV infection
CLL: Chronic lymphocytic leukemia
CR: Complete remission
CRP: C-reactive protein
CSF: Cerebrospinal fluid
CTLA-4: Cytotoxic T-lymphocyte-associated protein 4
DCs: Dendritic cells
ESHCC: Early-stage hepatocellular carcinoma
HCC: Hepatocellular carcinoma
HCV: Hepatitis C virus
HL: Hodgkin Lymphoma
HLA: Human leukocyte antigen
IFN-γ: Interferon-γ
ITP: Immune thrombocytopenia
mB7-H3^**+**^: Membrane B7-H3^**+**^
MCL: Mantle cell lymphoma
MDS: Myelodysplastic syndrome
MHC: Major histocompatibility complex
MM: Multiple myeloma
MMPI: Matrix metalloproteinase inhibitor
MMR: Macrophage mannose receptor
MPEs: Malignant pleural effusions
MPP: Mycoplasma pneumoniae pneumonia
MS: Multiple sclerosis
NKTCL: Natural killer/T-cell lymphoma
NPEs: Nonneoplastic pleural effusions
NSCLC: Non-small cell lung cancer
OS: Overall survival
PBMCs: Peripheral blood mononuclear cells
PD-1: Programmed cell death-1
PD-L1/2: Programmed cell death ligand-1/2, also known as B7-H1/2
RA: Rheumatoid arthritis
SAHA: Suberoylanilide hydroxamic acid
sCTLA-4: Soluble CTLA-4
sIL-2R: Soluble IL-2R
SLE: Systemic lupus erythematosus
SpA: Spondyloarthropathies
sPD-1: Soluble PD-1
SSc: Systemic sclerosis
T1DM: Type 1 diabetes mellitus
T2DM: Type 2 diabetes mellitus
TCR: T-cell receptor
TME: Tumor microenvironment
TNF-α: Tumor necrosis factor-α
